# An Electrochemical Screening Reactor Kit for Rapid Optimization of Electrosynthesis Applications

**DOI:** 10.1002/cssc.202402086

**Published:** 2024-12-20

**Authors:** Leon Wickert, Kevinjeorjios Pellumbi, Julian T. Kleinhaus, Jonas Wolf, Julia Obel, Rui Cao, Daniel Siegmund, Ulf‐Peter Apfel

**Affiliations:** ^1^ Activation of Small Molecules/Technical Electrochemistry Ruhr University Bochum Universitätsstr. 150 44801 Bochum Germany; ^2^ Department Electrosynthesis Fraunhofer Institute for Environmental, Safety and Energy Technology UMSICHT Osterfelder Str. 3 46047 Oberhausen Germany; ^3^ Key Laboratory of Applied Surface and Colloid Chemistry Ministry of Education, School of Chemistry and Chemical Engineering Shaanxi Normal University Xi'an 710119 China

**Keywords:** 3D printed reactor, Electrochemical hydrogenation, Electrochemical reactor, Electrochemistry, High-throughput screening, Industrial chemistry, Scalable electrosynthesis

## Abstract

Electrosynthetic processes powered by renewable energy present a viable solution to decarbonize the chemical industry, while producing essential chemical products for modern society. However, replacing well‐established thermocatalytic methods with renewable‐powered electrosynthesis requires cost‐efficient and highly optimized systems. Current optimization of electrolysis conditions towards industrial applications involving scalable electrodes is time‐consuming, highlighting the necessity for the development of electrochemical setups aimed at rapid and material efficient testing. To address this challenge, we introduce a 3D‐printed electrochemical screening reactor designed for rapid optimization of relevant electrochemical parameters, utilizing electrode and membrane materials comparable to those in scalable electrolyzers. The reactor comprises eight individual two‐compartment cells that can be operated simultaneously and independently. To evaluate the reactor′s ability to provide meaningful insights on scalable cell designs, trends were compared with data from conventional scalable systems for electrochemical hydrogenations (EChH), demonstrating fast and accurate parameter optimization with the screening reactor. A detailed description of the reactor design and construction data files are provided using open‐source tools, enabling easy modification for anyone. We believe this screening reactor will be a valuable tool for the scientific community, for facilitating the discovery of reactions with customized electrode designs and rapidly improving conditions in established large‐scale electrolyzers.

## Graphical abstract

A 3D‐printable electrochemical screening reactor kit enables rapid optimization of electrosynthesis parameters through eight independent two‐compartment cells. Utilizing industrially relevant electrodes, the ElectroHermes reactor provides transferable insights to scalable systems. The included open‐source design files allow for customization, promoting efficient condition optimization and accelerating the electrification of the chemical industry.

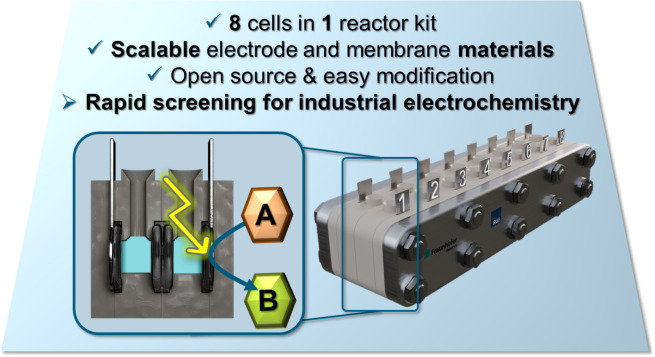

## Introduction

Electro‐organic reactions represent an emerging method for sustainable and safe synthesis routes for a broad variety of chemical compounds.[^1^, ^2^, ^3^, ^4^, ^5^, ^6^, ^7^, ^8^] This quality can be attributed to the utilization of electrons from renewable electricity as green redox reagents and the mild reaction conditions of electrosynthesis.^[9]^ Despite their benefits, electrochemical routes are rarely applied in industrial organic synthesis, where established thermocatalytic routes dominate large‐scale chemical production.[^7^, ^8^, ^11^] A reason for this gap is the challenge of transferring trends between the often‐employed H‐type cells and more industrially relevant flow‐cells that can compete with established thermochemical methods.^[15]^ Such developments require optimization in a cost‐efficient and rapid manner for a meaningful transition to electro‐organic systems to take place.^[16]^ However, various interacting process parameters and reactor components must beconsidered in such optimization processes, especially catalyst design, catalyst binder, electrode support, electrolyte composition, current density, fluid dynamics and cell geometry, among others.^[17]^ While all these variables can enable precise process control in electrochemical synthesis, their complexity makes the development of competitive electrochemical processes in the chemical industry utterly time‐ and cost‐intensive.^[20]^

In regard to this constant need for continuous tailoring and optimization, our group has recently demonstrated that optimization processes for scalable electro‐organic reactions are not only time‐ and material‐intensive but may need to be revised entirely for different starting materials, substrates as well as binder types.[^8^, ^21^, ^22^, ^23^, ^24^] Thus, establishing an accelerated and standardized experimentation process is imperative to approach the application perspective of electrosynthesis. To achieve this, high‐throughput screening reactors could be incorporated to speed up the data collection or provide large data sets for the development of machine learning models, ultimately accelerating parameter optimization.[^25^, ^26^]

High‐throughput screening has already been implemented in electrochemical applications. Depending on the respective application, reactors have been designed for liquid phase reactions in batch‐type[^25^, ^29^, ^31^] and flow‐type cells,[^32^, ^34^] as well as reactions with gaseous feedstocks.^[35]^ Most of the high‐throughput screening cells use metal electrodes in form of rods or plates in undivided reactors. While these electrodes can provide initial insights into intrinsic material activity, they might lack transferability to scaled‐up reactors with structured and tailored electrodes, such as zero‐gap electrolyzers.[^8^, ^37^] In addition to a mature electrode configuration, precise control and independent measurements are necessary to avoid interference between each individual cell, enable fast, error‐robust screening and to maximize flexibility of the developed system.[^31^, ^34^] These requirements imply a high flexibility and modifiability of the reactor fabrication. In recent years, highly customizable reactors for electrochemical purposes have been increasingly often manufactured *via* 3D printing techniques.^[39]^ This technology enables cheap prototyping of complex 3D structures from tailored Computer Assisted Design (CAD) models within a short amount of time and minimal material waste.

These above‐mentioned challenges and possibilities inspired us to design a reactor that closes the gap between rapid small‐scale screening and scaled‐up electrolyzer optimization. In this work, we present the design, guidelines as well as application examples of a 3D‐printed electrochemical screening reactor for rapid data acquisition that uses scalable electrodes in a divided setup. The reactor features eight individually controlled cells with a high electrode area‐to‐electrolyte‐volume ratio, ensuring fast conversion of reactants at high current densities and enabling an approximately 8‐fold faster experimentation time. We ensured the reproducibility among single cells and transferability of screening cell insights to previous results for electrochemical hydrogenation reactions in scalable zero‐gap setups. With an instructional assembly video and all relevant 3D design and construction files for custom modification being available in an open‐source repository, we envision that this reactor will become a valuable tool especially for the electrochemical community, enabling an accelerated data acquiring process for the optimization of scalable electrolyzers.

## Results

### Reactor Design

**Figure 1:**
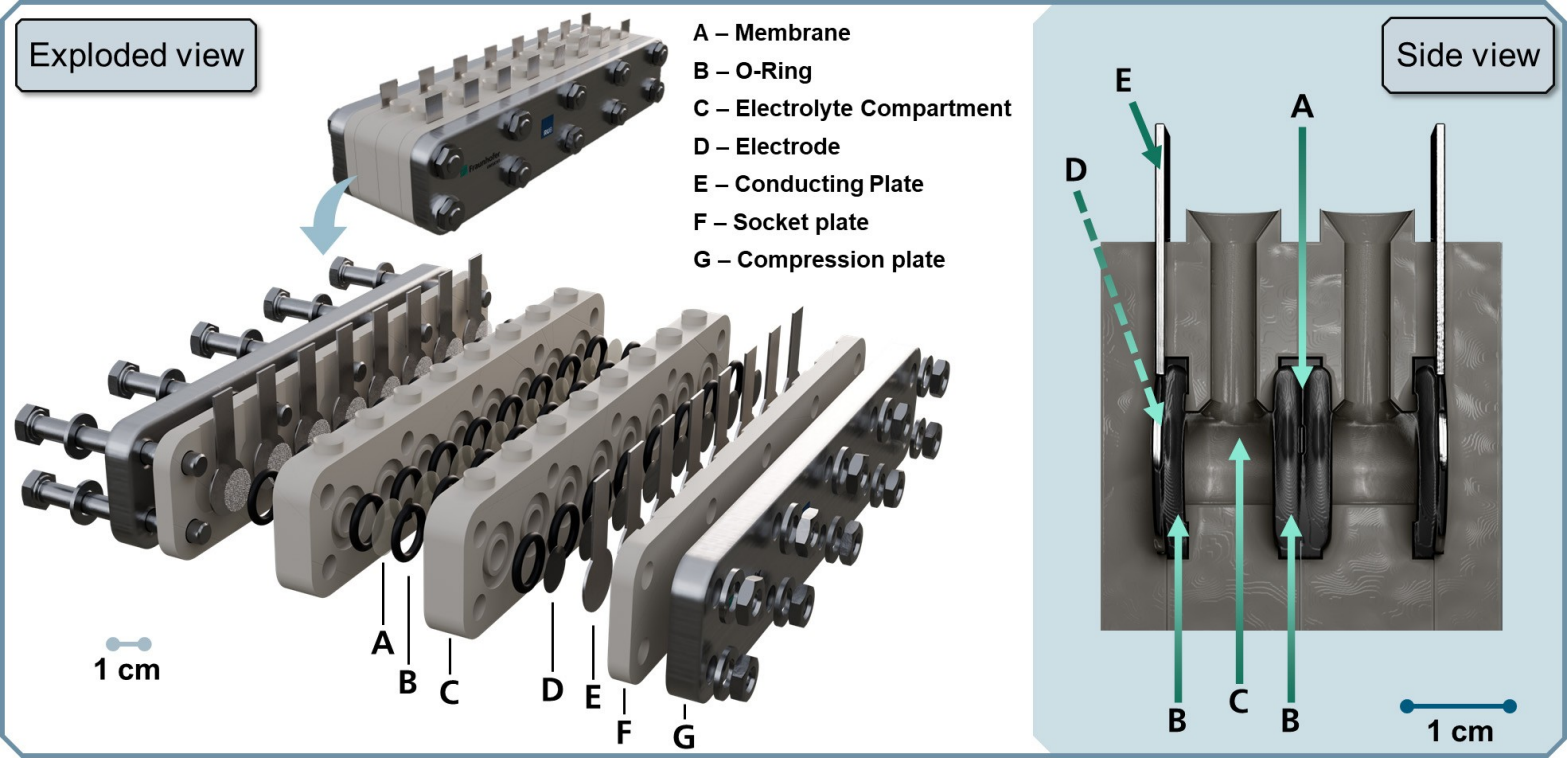
ElectroHermes reactor design. Exploded view (left) shows most important components of ElectroHermes. Side view (right) visualizes the H‐type structure of a single cell of the reactor.

The screening reactor which we call “ElectroHermes” consists of two socket plates (F) and two electrolyte compartment elements (C), forming eight separated H‐type cells (Figure 1). These parts can be manufactured with a minimum workload *via* 3D‐printing for rapid prototyping and quick adjustments based on experimental needs.^[43]^

A carbon fiber‐reinforced PET filament (PET CF) was used to fabricate 3D‐printed compartments for experiments in aqueous media. PET CF has shown excellent mechanical and chemical stability in various organic solvents (Table S2). However, it was observed that 3D‐printed parts tend to absorb organic solvents, such as methanol, between the individual material layers, which led to a partial loss of electrolyte volume. Therefore, solid poly(etherethylketone) (PEEK) blocks were milled *via* computerized numerical control (CNC) to form compartments and socket plates suitable for organic solvents. PEEK compartments were used for experiments with organic solvents while PET CF parts are fully suitable for electro‐organic experiments employing aqueous electrolytes and have been used for the respective experiments. Steel plates (G) were used to ensure homogeneous compression of all individual cells and avoid deformation of 3D‐printed parts. To enhance the mass transport of the reactants during electrolysis an orbital shaker was employed to agitate the electrolyte. The operating current was applied *via* a Landt Battery Testing System CT3002A‐10 V, and the electrical connection of each cell was made with a clamp rail, allowing for fast and simple connection and disconnection of the potentiostat (Figure S2). This design enables each cell to be operated simultaneously with individually controlled current density or voltage. To allow for custom modification of the reactor, all relevant design files including technical drawings, 3D constructions and models are provided by a link to an open‐source repository in the supporting information (SI). Moreover, to facilitate straightforward construction and operation, a demonstrational assembly video, fabrication details and a detailed description of reactor dimensions (Figure S1) can be found in the repository and the SI. The total cost of the screening reactor sums up to less than $ 250 without consumables such as electrodes, membranes and electrolyte. Due to the potentiostat making up the by far highest share in the total cost of the setup, an alternative low‐cost potentiostat is discussed in the “Expanding the application scope through customization” section.

### Reproducibility Evaluation

**Figure 2:**
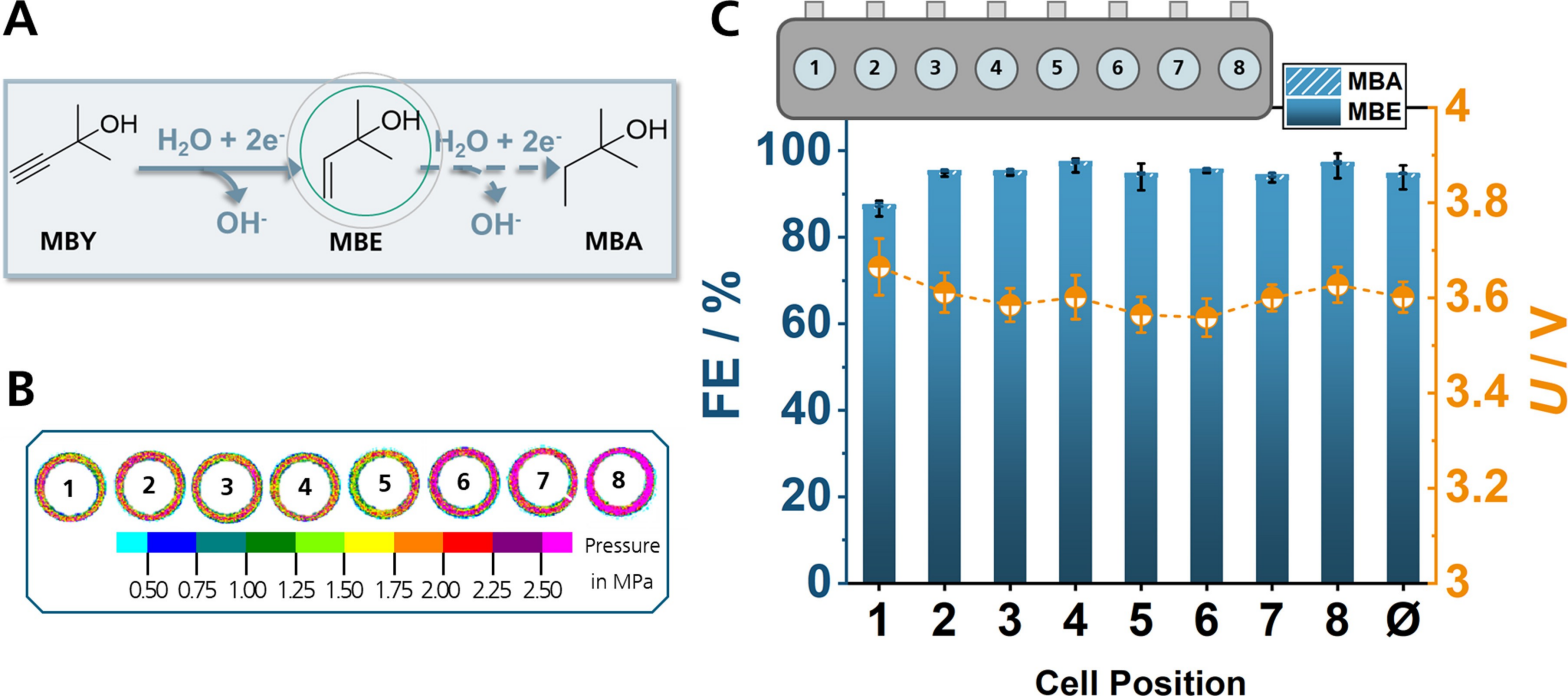
EChH of MBY to MBE with full hydrogenation to 2‐methylbutan‐2‐ol (MBE) as side reaction (A). Pressure test results for tightening all reactor bolts with 5 Nm (B) and faraday efficiencies and cell voltages of 2 distinct runs for each cell position (C) using 1 mg cm^−2^ Ag‐NPs, 1 M MBY in 0.3 M aqueous KOH at 20 mA cm^−2^.

To evaluate the reproducibility of each individual cell and the comparability of the cells among each other, eight identical experiments were performed in parallel with EChH of the vitamin A and E synthon 2‐methyl‐3‐butyne‐2‐ol (MBY) to 2‐methyl‐3‐butene‐2‐ol (MBE) as model reaction (Figure 2A).^[45]^ The oxygen evolution reaction (OER) was chosen as the anode reaction. However, any electrochemical reaction that can be performed in standard H‐type cells could be conducted with ElectroHermes. The reaction conditions and electrode fabrication are comparable to previously reported results in scalable and modular zero‐gap reactors, in which the two electrodes are mechanically pressed onto a micro‐thin ion exchange membrane.[^46^, ^47^] In regard to the employed catalysts, due to their high catalytic activity in our previous studies, silver nanoparticles (Ag‐NPs) spray‐coated on carbon paper were employed as cathodes, while compressed Ni foam was used as anodes. The catholyte consisted of 1 M 2‐methyl‐3‐butyn‐2‐ol (MBY), and 0.3 M KOH in water, while 2 M KOH in water was used as anolyte. A volume of 1 ml for catholyte and anolyte solution was used for all experiments and the reactor was placed on an orbital shaker at 160 rpm to promote convection. Each pair of half‐cells was separated by a Fumasep FAA‐3‐PK‐130 anion‐exchange membrane (AEM) to allow for OH^−^ migration. To enable fast conversion in a short amount of time, the EChH of MBY was performed at 20 mA cm^−2^ for 40 min (Figure 2).

Recent studies have shown that reactor compression influences cell voltage and faraday efficiency (FE) in EChH and electrochemical CO_2_ reduction (CO_2_R).[^21^, ^48^] Hence, we evaluated the compression effect in the ElectroHermes reactor, prior to other experimentation, by assembling the cell with different degrees of tightening, including a torque of 5 Nm on all 10 bolts, reduced torque on outer bolts as well as assembly with 4 bolts. (Figure 2B, Figure S4). Tightening all reactor bolts with a torque of 5 Nm, resulted in the smallest error in FE among the single cells, compared to other tightening modes, indicating that a high compression is beneficial for the reproducibility of results. Detailed results of pressure tests are provided in the SI. Even though pressure differences of the individual O‐Rings could be detected, no significant influence on the resulting FE could be observed, possibly due to the small pressure variation between the different O‐rings being in the range of 1 MPa. With the optimized compression mode, the Faraday efficiencies (FEs) and errors are comparable to previous EChH experiments with Ag‐NPs in a zero‐gap reactor (Figure 2A).^[46]^ In addition to pressure tests, the heat accumulation within the electrolyte chambers was monitored in the final minutes of the electrolysis. The electrolyte temperature of cell position 4 was found to be 0.2–0.3 °C higher than at cell position 1, indicating variations in heat dissipation between the cells. However, the difference in heat dissipation is not large enough to significantly influence the reproducibility of the experiments.

The crossover of products from the cathode compartment to the anode compartment did not surpass 3 % of the FE (Figure S9). Overall, FEs of 94±3 % and cell voltages of 3.60±0.03 V (Figure 2C, Figure S3) were obtained. The most significant deviation was observed at cell position 1 with an FE of 87 % and a cell voltage of 3.67 V (Figure 2C). This observation led us to rearrange the order of experiments in subsequent runs, so that the same conditions are measured in different cell positions to reduce systematic errors. To avoid cross‐contamination caused by electrowetting of the microporous catalyst layer of the electrodes and to prevent a decline of electrode performance over time, the reactor was disassembled after each experiment to replace the electrodes and clean the components. A detailed cleaning procedure can be found in the experimental section. Despite minor deviations, the reproducibility of the screening reactor within each cell was confirmed.

Regarding the number of conductible experiments when working with ElectroHermes, we noticed that the screening cell can be assembled, operated and disassembled at approximately the same rate as a single cell zero‐gap reactor, taking electrode and electrolyte preparation, as well as sample collection into account. Therefore, with the use of ElectroHermes, approximately seven additional single cell experiments can be performed in the same duration of a single cell zero‐gap reactor experiment.

### Comparability to a Scalable System

**Figure 3:**
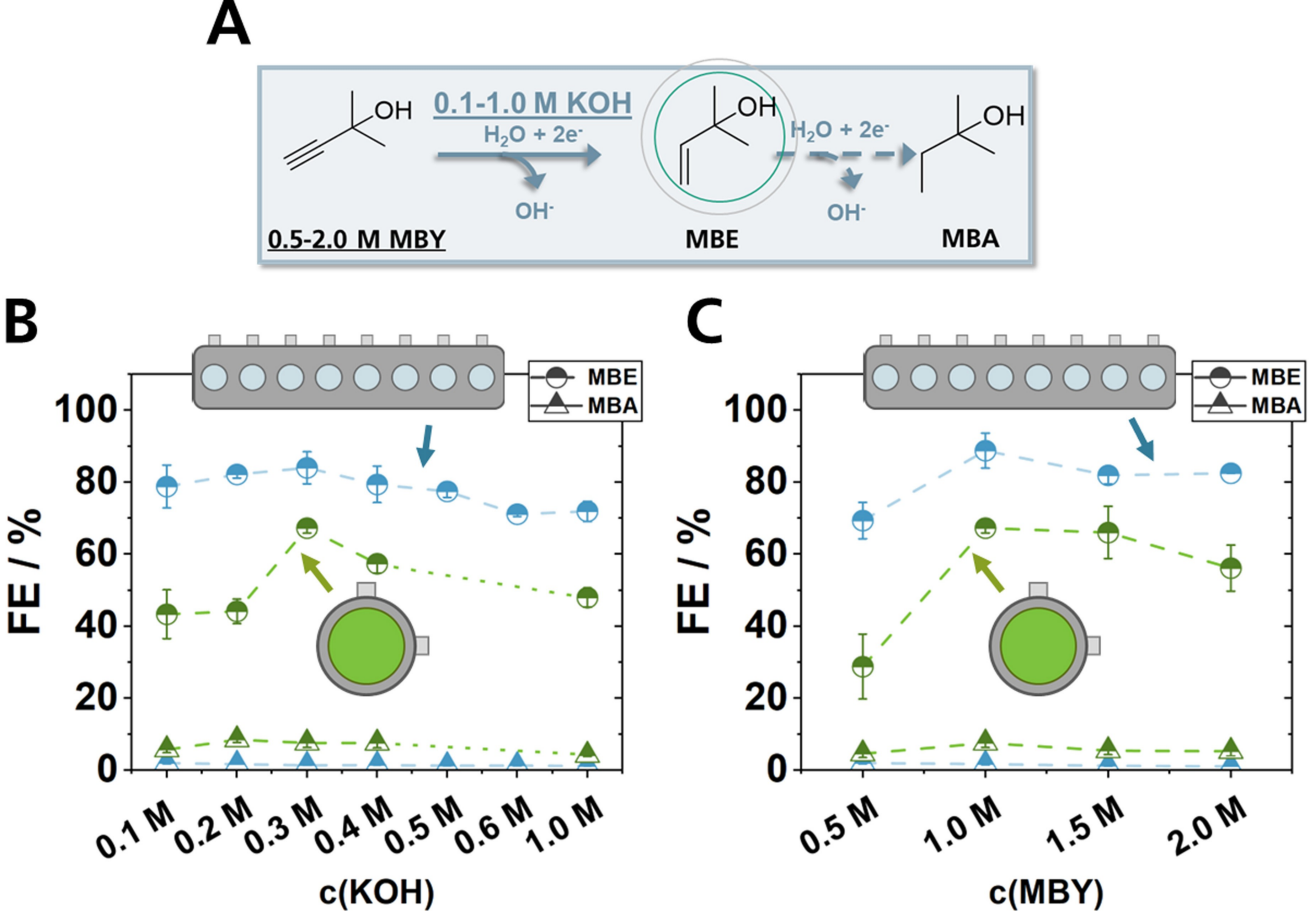
Comparison of FEs within ElectroHermes and a zero‐gap reactor for the EChH of MBY (A) with different concentrations of potassium hydroxide (B) and MBY (C) with 5 mg cm^−2^ Pn, 1 M MBY in 0.3 M aqueous KOH. KOH concentration studies in the screening reactor were performed in two trials with random variation of cell position for each repetition. MBY concentration studies were performed in one trial with each data point resulting from the cell position pairs 1–5, 2–6, 3–7, and 4–8 for 0.5 M, 1.0 M, 1.5 M and 2.0 M MBY, respectively. Zero‐gap experiments (green) were conducted with 50 ml catholyte solution at 80 mA cm^−2^ for 2 h, while ElectroHermes (blue) experiments contained 1 ml catholyte solution and were conducted at 20 mA cm^−2^ for 40 min.

To truly assess the transferability of optimized reaction conditions from ElectroHermes to a zero‐gap reactor, previously reported experiments on electrolyte and substrate concentrations for the EChH of MBY with the pentlandite (Pn) catalyst Fe_3_Ni_6_S_8_ in a zero‐gap reactor were investigated, this time using ElectroHermes with similar electrolyte and electrode configuration (Figure 3A).^[21]^ Here, we focus mainly on understanding if the overall trends between the different conditions are similar, since the distinct FE values may certainly vary in absolute numbers due to the different mass transport architectures of the two cell types and different reaction times as well as current densities (Table S3). Notably, to collect all data points for the electrolyte screening shown in Figure 3B, including replications, ElectroHermes was assembled only two times. In addition, the eight data points of the MBY concentration investigation were obtained with a single assembly of ElectroHermes (Figure 3C), highlighting the ability of ElectroHermes to generate whole data sets of electrochemical experiments, instead of single data points with conventional single cell reactors.

Overall, EChH in ElectroHermes leads to increased total FEs and a higher share of the preferred semi‐hydrogenated product, compared to experiments in a zero‐gap electrolyzer (Figure 3B, C), possibly due to a lower ratio of current density to substrate concentration, as previously observed.^[22]^ Interestingly, the trend in FE in the zero‐gap electrolyzer shows a significant increase from 0.2 M to 0.3 M KOH and decrease for concentrations above 0.4 M KOH, while this trend is not significantly pronounced for experiments with ElectroHermes between 0.1 M and 0.5 M KOH. However, the statistical significance of the results in ElectroHermes could be increased by screening at higher current density. Nevertheless, both cells demonstrate that the optimum KOH concentration for the EChH is most likely between 0.1 M and 0.5 M for reactions with ElectroHermes, closely matching the optimum of 0.3 M in a zero‐gap cell. While measurements without conducting salt did not severely effect cell voltages in the zero‐gap electrolyzer due to the minimized ohmic resistance, the intrinsically higher ohmic resistances in the H‐type cells of ElectroHermes hamper measurements with low conductivity electrolytes. Additional pH measurements before and after electrolysis indicate a slight increase of catholyte pH from 13.8 to 13.9, possibly originating from hydroxide transport from the 2 M KOH anolyte solution before the start of the electrolysis and hydroxide generation due to electrolysis. Likewise, the anolyte pH decreased from 14.5 to 14.4 due to hydroxide consumption during OER. These observations are in accordance with our previous pH investigations in zero‐gap systems.^[21]^

Regarding the effect of the organic substrate concentration, both reactors show a significant increase in FE when the MBY concentration is raised from 0.5 M to 1.0 M (Figure 3C), previously attributed by us to a better mass transport of MBY to the electrode surface, favoring the EChH. Interestingly, a stagnation of FE is observed for both reactor setups, when the MBY concentration is increased to 1.5 M and 2.0 M, possibly due to a saturation of active centers on the electrode surface. These observations indicate that, despite their different cell architectures, performance trends of ElectroHermes could become guidelines for the zero‐gap reactor in the investigated electrolysis time frame.

The result of conducting the EChH of MBY at different KOH and MBY concentrations indicate that ElectroHermes is a convenient choice for rapidly optimizing electro‐organic reactions at an early development stage, e. g. to narrow down the range of optimal electrolyte and substrate concentration. These valuable insights could be adopted for larger‐scale systems and therefore accelerate electrochemical process development. Yet, to gain information on the influence of electrolyte flow rates or long‐term stability of electrodes, other suitable reactor designs for more advanced process parameters must be considered.

### Broadening the Application Scope for EChH

**Figure 4:**
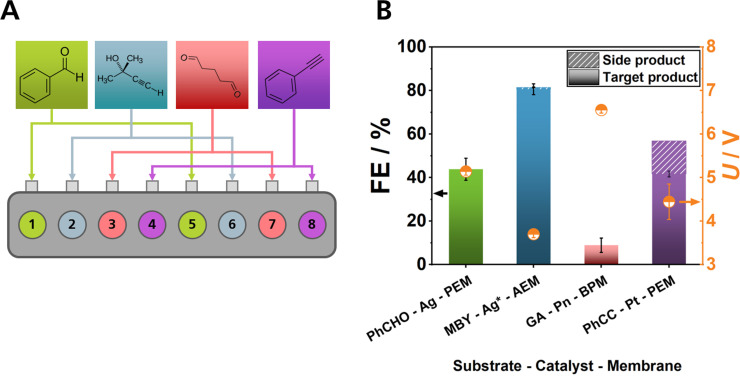
Exemplary EChH reactions in the ElectroHermes reactor (A) with various conditions (Substrate–Electrode–Membrane) (B) and their corresponding FEs (left black axis) and cell voltages (right orange axis). Experiments were performed in one trial with each data point resulting from the cell position pairs 1–5, 2–6, 3–7, and 4–8 for benzaldehyde (PhCHO) with 2 mg cm^−2^ Ag, MBY with 2 mg cm^−2^ electroplated Ag (Ag*), glutaraldehyde (GA) with 2 mg cm^−2^ Fe_3_Ni_6_S_8_ and phenylacetylene (PhCC) with 1 mg cm^−2^ Pt/C, respectively. Carbon paper was used as electrode support.

Beyond screening only electrolyte variations, an impactful screening cell design must also allow for the simultaneous conduction of experiments at an extended substrate scope, e. g. to test the activity of one catalyst for multiple organic substrates or vice versa. Especially for organic substrates that are not soluble in aqueous solutions, organic solvents must be employed, limiting the use of most anion‐exchanging membranes, due to their insufficient chemical stability in organic solvents. To highlight the application scope of the ElectroHermes reactor, EChH was conducted with various combinations of substrates, catalysts and membranes, which were previously investigated by our group in zero‐gap setups.[^23^, ^24^, ^46^] These included hydrogenation of phenylacetylene to styrene and hydrogenation of benzaldehyde to benzylalcohol with a PEM, glutaraldehyde hydrogenation to 1,5‐pentanediol and 5‐hydroxypentanal with a Fumasep FBM PK bipolar membrane (BPM) and silver‐electroplated electrodes (Ag*) as an alternative electrode choice for the EChH of MBY to MBE in an AEM setup (Figure 4A).^[46]^ For the EChH of benzylalcohol and phenylacetylene, methanol was used as organic solvent, underlining that ElectroHermes can be a valuable tool in the field of synthetic organic electrochemistry. In total, four experiments with their respective repetitions were conducted in a single trial. Due to the use of methanol as organic solvent for the PEM setups, PEEK compartments and socket plates were used, as discussed in the reactor design section.

In all individual cells the respective product was obtained and no crossover between individual cells is observed (Figure 4B, Figure S5, S6, S7, S8). With a Nafion 117 proton exchange membrane (PEM) setup, a FE of approximately 50 % is achieved for EChH products of both benzaldehyde and phenylacetylene, which is consistent with previous findings in zero‐gap cells.[^23^, ^46^] The use of a PEM also led to a more significant pH change in the catholyte from 14.2 to 14.5 due to the accumulation of hydroxide in the catholyte chamber that was previously balanced by anion transport through an AEM. The pH change from 14.5 to 14.4 in the anolyte solution of the PEM setup is similar to the AEM setup mentioned above. In contrast, increased cell voltages and lower FEs were observed for the EChH of glutaraldehyde in a BPM setup. Despite the elevated cell voltages in PEM and BPM configurations, the heat dissipation was comparable to the AEM setup mentioned previously, with a maximum temperature difference of 0.3 °C between cell position 1 and cell position 4. The EChH of aldehydes is often challenging due to pH dependency of side reactions, which could cause the relatively high errors coupled with low FE, making the interpretation of potential optimization results challenging.[^24^, ^49^] These values are, again, highlighting the limitations of current BPM generations.^[24]^ Further insights are needed to reduce errors and enable acquisition of valuable data for employing ElectroHermes in a BPM system.

### Current Density Screening

**Figure 5:**
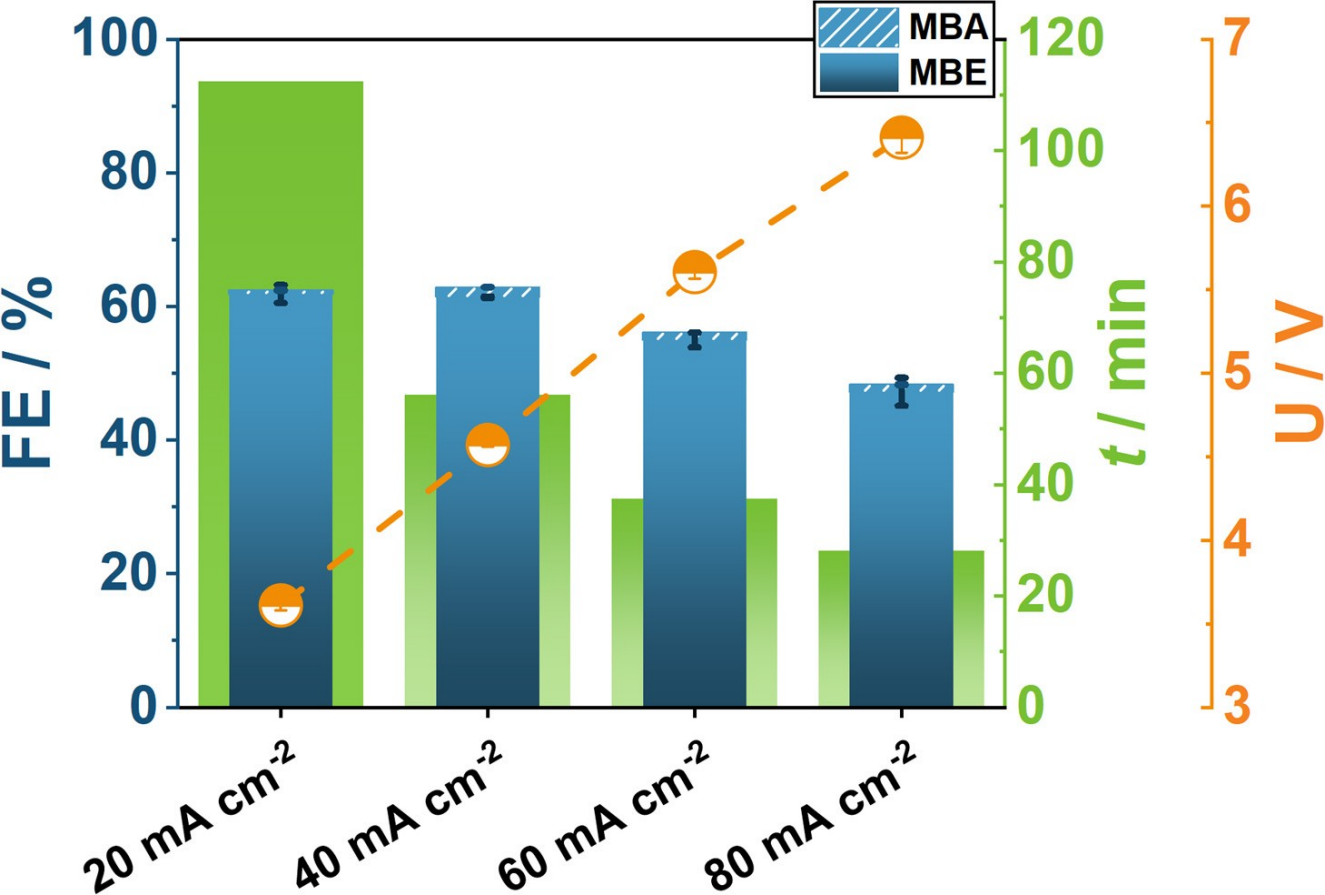
Current density investigation of the EChH of MBY with ElectroHermes reactor using 1 mg cm^−2^ Ag‐NPs on carbon paper, 1 M MBY in 0.3 M aqueous KOH. Experiments were performed in one trial with each data point resulting from the cell position pairs 1–5, 2–6, 3–7, and 4–8 for 20 mA cm^−2^, 40 mA cm^−2^, 60 mA cm^−2^ and 80 mA cm^−2^, respectively.

All experiments described above were performed at 20 mA cm^−2^ for 40 min. Higher current densities decrease the experimentation time and allow for a higher throughput of experiments, while providing results at more industrially relevant conditions. Thus, the EChH of MBY was investigated with a range of increasing current densities and the reaction time adjusted accordingly, so that the same charge was transferred in each experiment. Hence, FE at 20 mA cm^−2^ are decreased compared to the shorter reaction times in the previous chapters. Again, a single trial with ElectroHermes was required to obtain all data points shown in Figure 5.

The results indicate that even at elevated current densities of up to 80 mA cm^−2^ only a small decrease in FE is present, which can be attributed to mass transport limitations (Figure 5). Cell voltages increased from 3.6 V at 20 mA cm^−2^ to 6.4 V at 80 mA cm^−2^. The high cell voltage can be attributed to the intrinsically high ohmic resistance due to the inter‐electrode gap and the contribution of the sluggish OER. With regard to elevated current densities, ElectroHermes can provide a realistic insight into electrode activity of electrosynthetic processes. However, possible future improvements of the cell design could focus on reducing the inter‐electrode distance and implementing continuous electrolyte flow to allow for measurements at higher current densities.

### Expanding the Application Scope Through Customization

Due to the modularity of the reactor design, the system can be upgraded and modified to widen the application scope and serve specific needs of users. For example, O‐rings may be exchanged with flat gaskets to allow for a straightforward variation of electrode size and a finetuning of cell compression, while clamps or a bench vise could be incorporated to simplify reactor assembly, as described in other high‐throughput systems.^[50]^ We also included additional reactor caps with a small hole for electrolysis with highly volatile solvents and without a hole for storing purposes after electrolysis in the open‐source repository. Furthermore, a reference electrode could be incorporated into the current design to provide information in half‐cell potentials and to include a larger range of applicable electrochemical characterization techniques. A suitable adapter for a Gaskatel Mini‐Hydroflex RHE is provided in the open‐source repository. This adapter serves as an example and could be modified to incorporate other reference electrodes. Likewise, additional tube connections could be added to the existing design to enable flow electrochemistry with suitable pump systems. To reduce the high cost of commercial (multi‐channel) potentiostats that would surpass the ElectroHermes cost of $ 250 by far, a USB Potentiostat/Galvanostat published by Dobbelaere *et al*. could serve as a convenient substitute with hardware costs of less than $ 100 and a wide range of potential and current (±25 mA at ±8 V).^[52]^

## Conclusions

Herein, we present an affordable electrochemical multi‐cell screening reactor that enables rapid optimization of components and parameters for electrosynthetic applications in the lab‐scale whilst using electrodes and membranes aimed for large‐scale electrolysis application. With an eight‐cell‐in‐one reactor design approach, the screening setup allows for the generation of complete data sets by screening multiple different electrolysis conditions and components at once in an independent manner, enabling an eightfold increase in data acquisition volume. Through application examples in the area of electrochemical hydrogenations we are able to show that trends in the screening reactor regarding the effect of electrolyte and substrate concentration on the EChH of 2‐methyl‐3‐butyne‐2‐ol can be reproduced from previous results in a zero‐gap cell, highlighting the ability of the “ElectroHermes” screening setup to quickly optimize reaction conditions and accelerate early process development for industrially relevant electrolysis. A variety of applications within the field of EChH, including measurements in aqueous and organic media, using AEM, PEM and BPM setups with different organic substrates and investigation of elevated current densities has furthermore proven the versatility of the screening reactor. Finally, we provide detailed information on the reactor design and fabrication, allowing other electrochemists in the field of electrosynthesis to modify the reactor according to their needs. We believe that the “ElectroHermes” screening reactor can serve as a valuable tool for electrosynthetic applications, facilitating the rapid discovery of new electrochemical reaction routes or the optimization of parameters for various applications.

## Experimental

### Materials

The PTL consisted of H24 (Freudenberg) carbon paper and Fumasep® FAA‐3‐PK‐130 membrane and Fumasep FBM (FUMATECH BWT) were used as AEM and BPM respectively, while N117 (Ion Power) was used as PEM. Ni foam (Goodfellow) was used as anode material. As binding material, PTFE dispersion (QuinTech) and Knyar Flex Ultraflex B® PVDF (Arkema) was used. All other employed chemicals were purchased from commercial suppliers and used without further purification. The pentlandite catalyst Fe_3_Ni_6_S_8_ was synthesized by mechanochemical means as described previously.[^22^, ^53^]

### Electrode Fabrication

The employed cathodes were fabricated comparable to previous protocols.[^21^, ^22^, ^46^] For the silver electrodes, 0.5 g of Ag‐NPs were mixed with 25 ml methanol and 1,11 g of a 5 % PVDF solution in methanol. The mixture was ultrasonicated for 5 min. The ink was stirred during the following air‐brushing process. With an Iwata SBS airbrush, the suspension was homogeneously spray‐coated on a 8.5x8.5 cm H24 carbon paper that was heated to 100 °C until the desired catalyst loading was achieved. The final loading was calculated from the weight difference of the electrode sheet before and after spray‐coating. The resulting electrode sheets were cut into circular electrodes of 15 mm diameter. For the Pn containing electrodes, 0.5 g of Fe_3_Ni_6_S_8_ with 15 g 2‐propanol, 4 ml H2O and 0.2 g Triton X‐100 were mixed. After the mixture was placed in an ultrasonic bath for 5 min, the ink was dispersed for 1 min at 13,600 rpm using a T18 digital Ultra–Turrax (IKA‐Werke GmbH & CO. KG). Afterwards, 61.8 μL of a solution of 60 wt.% PTFE was added to the ink. The spray‐coating process was performed as described above. The PTFE‐containing electrodes were sintered at 260 °C for 20 min to remove the surfactant.

### Electrochemical Screening

The electrochemical measurements were conducted in an in‐house built 8‐cell reactor. Compressed Ni foam was used as anode while spray‐coated H24 carbon paper was used as cathode. The electrodes and membranes are held in place with O‐rings. Membranes were conditioned in the anolyte solution over‐night prior to electrolysis. The cell was compressed with additional steel end plates with multiple compression steps (2.5 Nm, 5 Nm, 5 Nm), with inner bolts being compressed first and outer bolts last (Figure S4C). An instructional assembly video is provided in the open‐source repository that can be found in the SI. For each cell, 1 ml of electrolyte was used. Electrolyte convection was enhanced by using a BioSan Orbitalshaker PSU‐10i at 160 rpm. The operating current was applied *via* a Landt Battery Testing System CT3002A‐10 V with eight individual channels. The electrical connection was realized with clamps connected to the titanium conducting plates. Each experiment was conducted for 40 min. New membranes and cathodes were used for each experiment. The cleaning procedure included complete disassembly of the reactor and removing the electrodes. Subsequently, all compartments in contact with electrolyte were rinsed with water and ethanol, including electrolyte compartments, conducting plates, socket plates, O‐rings and Ni foam anodes. More persistent contaminations were removed with brushes or pipe cleaners. In addition, to remove possible electrode residues from O‐rings, the O‐rings were cleaned by ultrasonication in a 1 : 1 mixture of water and ethanol for 10 min. Each experiment was repeated at least twice to ensure reproducibility. Repetition experiments were performed with each individual experimental condition being investigated in another, randomly chosen cell position to reduce systematic error.

## Conflict of Interests

The authors declare no conflict of interest.

1

## Supporting information

As a service to our authors and readers, this journal provides supporting information supplied by the authors. Such materials are peer reviewed and may be re‐organized for online delivery, but are not copy‐edited or typeset. Technical support issues arising from supporting information (other than missing files) should be addressed to the authors.

Supporting Information

## Data Availability

The data that support the findings of this study are available in the supplementary material of this article. Additional data and materials can be accessed at Open Science Framework (OSF) via DOI: 10.17605/OSF.IO/K268H (https://osf.io/k268h/).
