# Supplementary material for: An Electrochemical Screening Reactor Kit for Rapid Optimization of Electrosynthesis Applications
Source: ChemSusChem. 2024 Dec 20;18(8):e202402086. doi: 10.1002/cssc.202402086 (PMC11997912; doi:10.1002/cssc.202402086)
Supplement: Supplementary file 1 — Supporting Information [file CSSC-18-e202402086-s001.pdf]

ChemSusChem

Supporting Information

An Electrochemical Screening Reactor Kit for Rapid Optimization of Electrosynthesis Applications

Leon Wickert, Kevinjeorjios Pellumbi, Julian T. Kleinhaus, Jonas Wolf, Julia Obel, Rui Cao, Daniel Siegmund,* and Ulf-Peter Apfel*

Supporting Information

An Electrochemical Screening Reactor Kit for Rapid Optimization of Electrosynthesis Applications

Leon Wickert[1,2], Kevinjeorjios Pellumbi[2], Julian T. Kleinhaus[1], Jonas Wolf[1,2], Julia Obel[2], Rui Cao[3], Daniel Siegmund[1,2]*, Ulf-Peter Apfel[1,2]*

Author affiliations

[1]Ruhr University Bochum, Activation of Small Molecules/Technical Electrochemistry, Universitätsstr. 150, 44801 Bochum, Germany.

[2]Fraunhofer Institute for Environmental, Safety and Energy Technology UMSICHT, Department Electrosynthesis, Osterfelder Str. 3, 46047 Oberhausen, Germany.

[3]Key Laboratory of Applied Surface and Colloid Chemistry, Ministry of Education, School of Chemistry and Chemical Engineering, Shaanxi Normal University, Xi'an 710119, China

Email corresponding author: daniel.siegmund@umsicht.fraunhofer.de & ulf.apfel@rub.de

ORCID iDs of the authors

Leon Wickert https://orcid.org/0009-0005-6016-1492

Kevinjeorjios Pellumbi https://orcid.org/0000-0002-5313-5799

Julian T. Kleinhaus https://orcid.org/0000-0002-0901-4789

Rui Cao https://orcid.org/0000-0002-1821-9583

Daniel Siegmund https://orcid.org/0000-0003-2476-8965

Ulf-Peter Apfel https://orcid.org/0000-0002-1577-2420

Open data repository

Additional data and materials can be accessed at Open Science Framework (OSF) via DOI: 10.17605/OSF.IO/K268H (https://osf.io/k268h/).

The open-source data repository includes an instructional assembly video of the ElectroHermes reactor, details on 3D-printing parameters, all required 3D model files (.stl) to reproduce the

reactor, CAD file (.f3d) for straightforward custom modification, CNC files (.html, .simpl) for machining PEEK components and technical drawings.

Reactor components and assembly

Table S1: List of reactor components, amount, cost and material source for fabrication of the screening reactor. Consumable materials (electrodes, membranes, electrolyte) are excluded.

#	Component	Amount	Material source
**1**	Steel Plate (water jet cutted, 1.4571)	2	CUT Wasserstrahlschneidtechnik
**2**	Titanium Plate (water jet cutted, 3.7035)	16	CUT Wasserstrahlschneidtechnik
**3**	O-Ring FKM75 (13.50 x 3.00 mm)	32	HUG Technik und Sicherheit
**4**	Filament Ultimaker PET CF (2.85mm)	362g	IGO3D
**5**	Bolt M8 x 80 mm A2	10	frantos
**6**	Nut M8 A2	10	frantos
**7**	Washer M8 A2	20	frantos
**9**	Connection clamps (optional)	8	Landt Instruments

Reactor fabrication and assembly

The screening reactor features eight individual divided cells, while each cell consists of two identical half-cells separated by an ion-exchange membrane (A) with a diameter of 19 mm. PET CF was chosen as 3D printing filament as soaking in water, acetone, methanol, 1-propanol, *n*-pentane, toluene, acetonitrile, 2 M KOH and 1 M H_2SO_4 for 72 h did not change its appearance, bending behavior (tested by hand), or weight significantly. Only in DCM, the filament became slightly more flexible and soaking in DMF or DCM led to an increase in weight by 14% and 24%, respectively.

Table S2: Weight difference of 10 mm PET CF filament (⌀2.85 mm) samples after soaking in various solvents or solutions for 72 h.

**Solvent**	**PET CF weight difference / %**
H2O	0,0
Aceton	6,4
MeOH	-0,8
1-Propanol	0,0
n-Pentan	-0,9
Toluene	0,5
Acetonitrile	3,6
DMF	13,7
DCM	23,9
2M KOH	-2,2
1M H_2SO_4	0,7

Each half-cell contains a titanium conducting plate (E) that is incorporated into the PET CF socket plate (F) element, facilitating the electrical connection of the electrode (D). Electrodes, e.g. carbon paper, carbon cloth, nickel foam or titanium mesh were cut to circles of 15 mm diameter, having an active geometric surface area of approximately 1,43 cm^2 after compression by fluoroelastomer (FKM) O-ring gaskets (B), which simultaneously seal the compartments to prevent leakage. To enable a fast and accurate placement of electrodes, O-rings and membranes, a template was 3D printed to hold the individual components in place during assembly (Figure SX). The electrolyte compartment covers a volume of 1.1 ml, which can be adjusted by changing the compartment cavity dimensions. Each electrolyte cavity has a hole on the top side that serves as gas outlet and enables straightforward filling with liquids using standard pipettes. A detailed list of reactor components, amount and source of materials is shown in Table S1. Steel compression plates (G) and titanium conducting plates (E) Figure S1 were water jet cut by local companies, while FKM O-Rings (B) are commercially available in a variety of sizes (here 13,5 mm (Inner diameter) x 3 mm (ring diameter)). Chemically and mechanically stable carbon fibre reinforced PET (Ultimaker PET CF black) was used as 3D printing filament for electrolyte compartments (C) and socket plate parts (F). However, other filaments (e.g. PP, ASA, PCTG, ABS, etc.) might be suitable, depending on the specific

application and its chemical and physical requirements. The parts were 3D printed using an Ultimaker S5 3D printer. Details for slicing (support material and configuration, print cores, temperatures, infill, etc.) are shown in Table SX. Overall, the printing process takes 23 h for all 4 parts. After removing possible PET CF residues, the finished parts can be used without further post processing. Alternatively, a CNC protocol can be used to machine these parts from PEEK or other suitable materials. The individual parts are assembled as shown in Figure 1A. If the tolerance of the titanium plate mount is too high, a small amount of super glue can be used to keep the titanium plates in place. A maximum of 10 M8x80 mm bolts with suitable nuts and washers are used for an equal compression of all compartments.

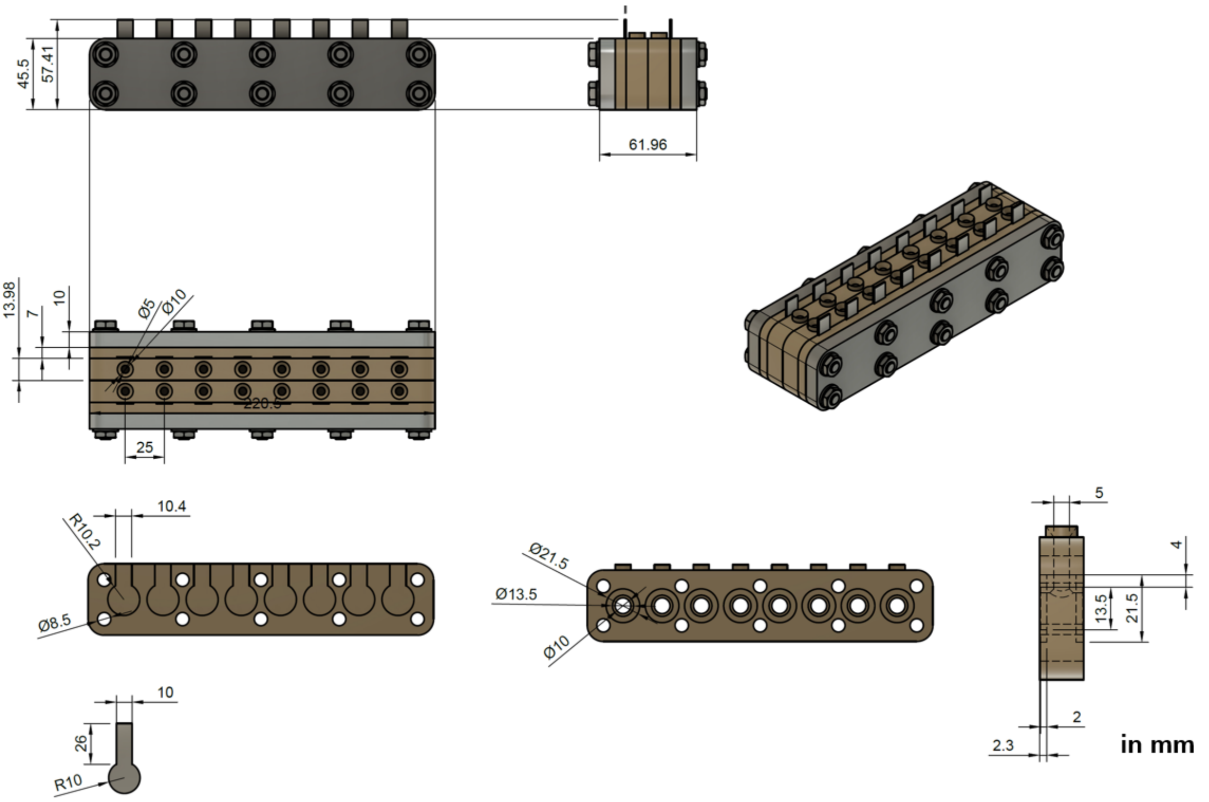

Figure S1: Technical drawing of all relevant cell components with respective dimensions in mm.

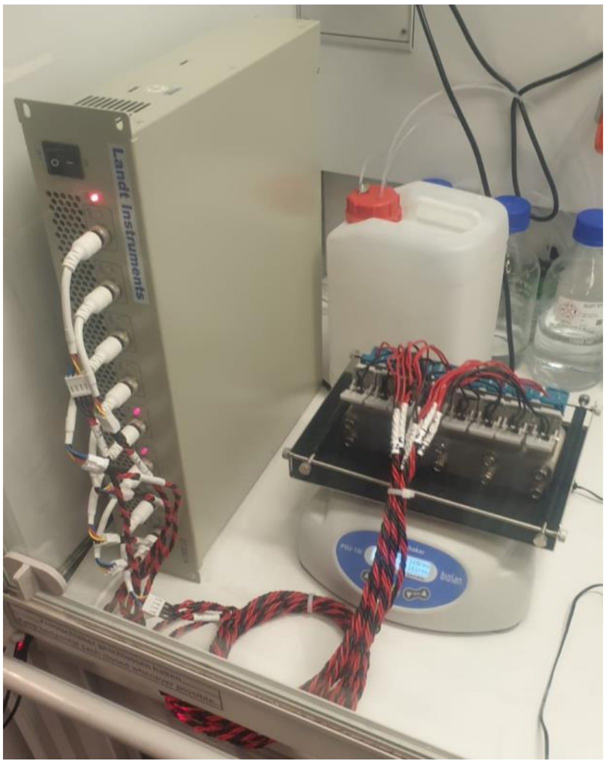

Figure S2: Photograph of ElectroHermes setup with orbital shaker and multichannel potentiostat connected to the screening electrolyzer with rail clamps.

Voltage trends

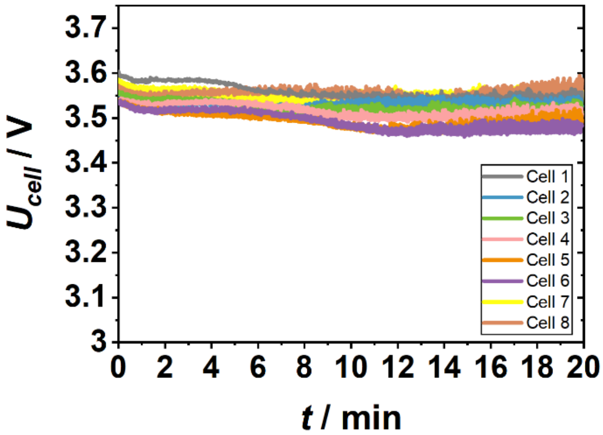

Figure S3: Cell voltage trends for each single cell of the EChH of MBY with Ag as catalyst from reproducibility tests.

All voltage curves show a similar trend which indicates a conditioning process of electrodes and membranes at the first 10 min of the experiment before stabilizing at a constant value, fluctuating by ±0.05 V in the last 10 min of the experiments.

Pressure tests with average pressures on each cell

To optimize the reactor assembly towards a homogeneous electrode compression, the cell was assembled with pressure sensitive paper placed between titanium conducting plates and electrodes. The reactor compression was altered by changing the tightening force of the respective bolts and the bolt position. Due to the smaller compression area of the outer bolts, three assembly strategies were investigated (Figure S4).

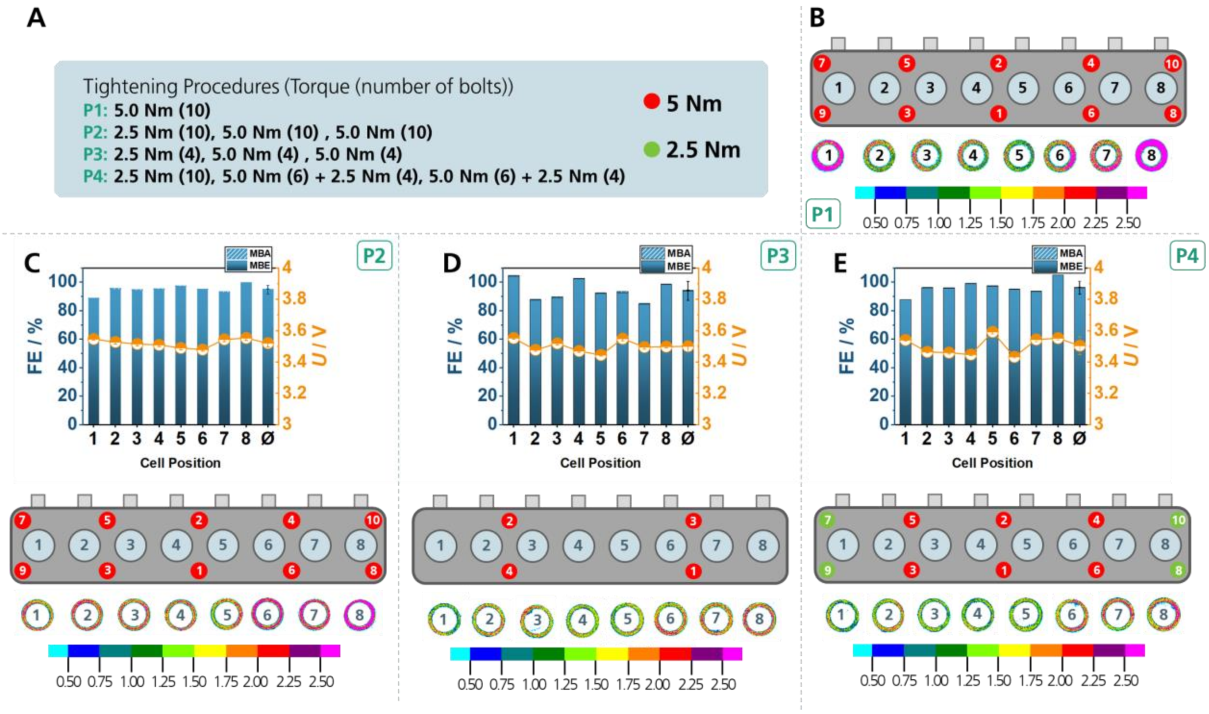

Figure S4: Tightening procedures (P1-P3) are displayed in A with respective torque. The number of bolts for this torque are shown in brackets, while multiple torques separated by commas stand for multiple tightening steps. The order of bolt tightening is indicated by the numbers at the respective bolt position. Faraday efficiencies and pressure tests of different compression modes for ElectroHermes are displayed in C-E in the EChH of MBY.

Tightening the bolts only once with a force of 5 Nm results in an inhomogeneous pressure distribution as shown in Figure S4B.

Compression tests with pressure sensitive paper indicated a slightly higher compression of cell positions six to eight, despite multiple tightening steps (Figure S4C-E). The average pressure for eight cells was 1.74±0.22 MPa (Figure S4C), 1.42±0.16 MPa (Figure S4D) and 1.35±0.19 MPa (Figure S4E), respectively. However, no clear trend between compression and FE could be determined among the individual cells in one trial. However, tightening with a higher torque could possibly improve the reproducibility of results. Due to the small error of assembly A, this tightening mode was used throughout the manuscript.

Comparison of ElectroHermes and zero-gap conditions

Table S3: Comparison of reaction parameters of ElectroHermes and zero-gap experiments

Parameter	ElectroHermes	Zero-gap
**Catalyst Support**	H24	Sigracell GFD 2.5 EA
**Current density**	20 mA cm^{-2}	80 mA cm^{-2}
**Operation mode**	Batch	Semi-batch
**Convection promoter**	Orbital shaker	Flow field
**Electrode area**	1.43 cm^2	12.57 cm^2

Product quantification and calculations

Product quantification was done *via* ^{1}H-NMR spectroscopy with potassium hydrogen phthalate as internal standard (S). For the ^{1}H-NMR analysis, 50 µL of the sample solution, 50 µL of a 1.25 µmol potassium hydrogen phthalate containing solution and 400 µL D_2O were mixed and measured in a Avance III 400 MHz spectrometer with 128 cycles at approximately 22 °C. For samples in organic solvents, deuterated trimethylsilylpropanoic acid sodium salt was used as standard and d_6-DMSO as solvent. The Faraday efficiency was calculated with following equation (n_P as amount of product p in mol, z as number of transferred electrons (2 or 4), F as Faraday constant (96485 A s mol^{-1}), i as applied absolute current in A and t as reaction time.

$$F.E._p = n_p \cdot \frac{zF}{i \cdot t} \cdot 100\ \%;\ [F.E._p] = \% \tag{S1}$$

The amount of product n_p was calculated with following equation (with I as the integral of the product peak in the ^{1}H-NMR spectrum normalized to the peak integral of the internal standard potassium hydrogen phthalate, R as the ratio of the number of hydrogen atoms assigned to the product peak and number of hydrogen atoms assigned to the standard peak and n_{St} as the amount of internal standard in the NMR sample (1.25 µmol)):

$$n_p = I \cdot R \cdot n_{St} \cdot 1000;\ [n_p] = mol \tag{S2}$$

The yield was determined with following equation:

$$yield = \frac{n_p}{n_{MBY}} \cdot 100\ \%;\ [yield] = \% \tag{S3}$$

The cell voltage values have been calculated by determining the arithmetic mean of the respective voltage data for all data points of the last 10 min of the experiment for both measurements. The arithmetic mean and the standard deviation of the resulting values was determined as the final data point used in the cell voltage axis.

Despite using different substrates within a single trial, no crossover between each individual cell could be observed in the ^{1}H-NMR spectra (Figure S5-S7). Furthermore, only traces of reaction products could be found in the anolyte solution for AEM experiments (Figure S8). For PEM and BPM measurements, no crossover of products could be observed).

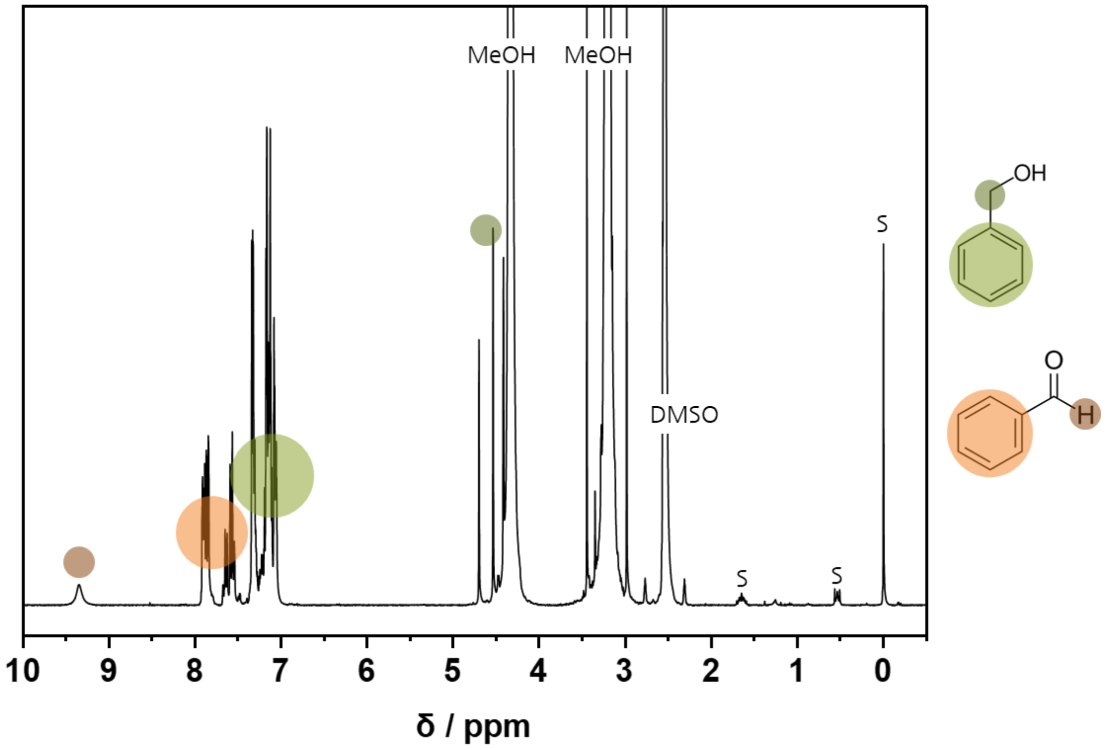

Figure S5: Representative ^{1}H-NMR spectrum of the reaction mixture after EChH of benzaldehyde after baseline correction and peak deconvolution.

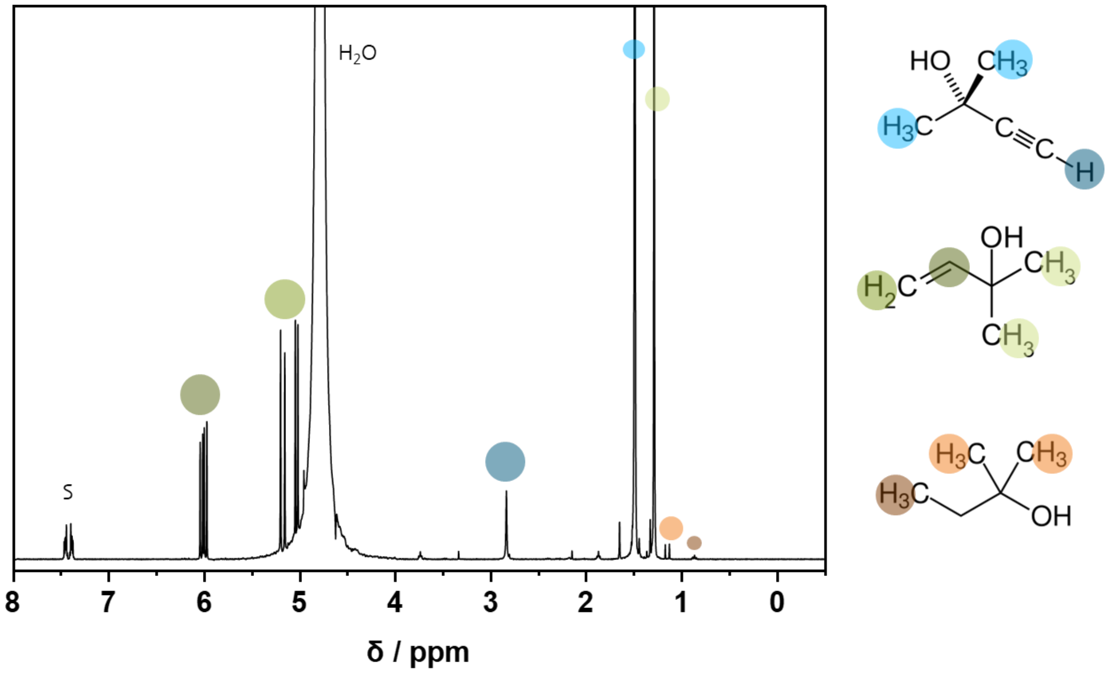

Figure S6: Representative ^{1}H-NMR spectrum of the reaction mixture after EChH of MBY after baseline correction and peak deconvolution.

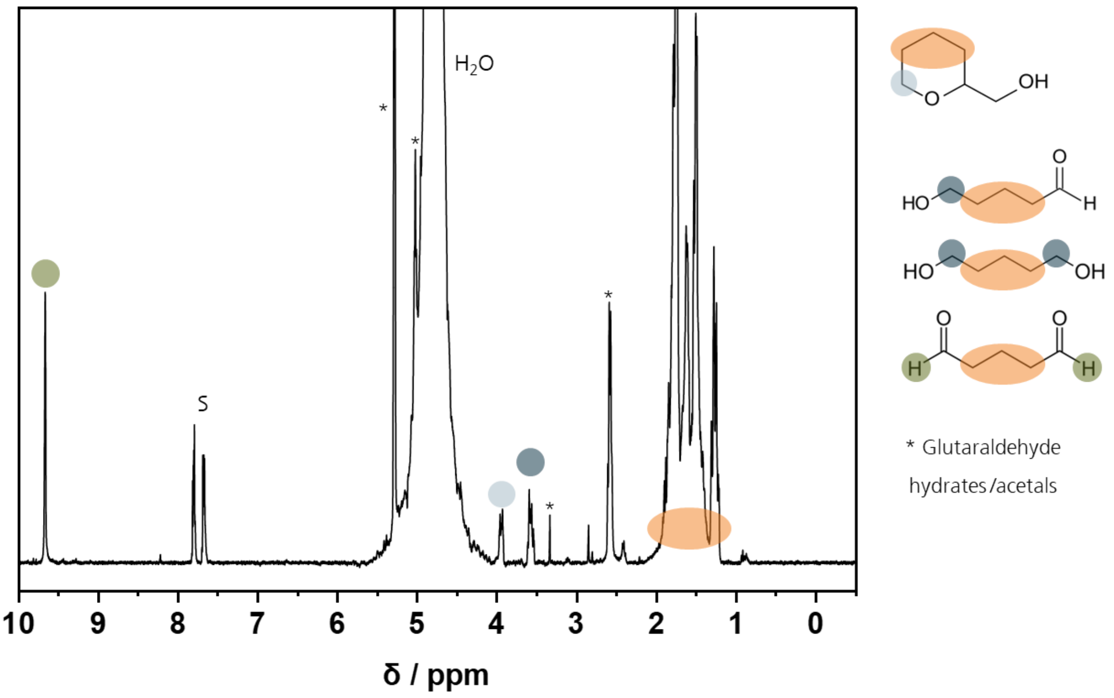

Figure S7: Representative ^{1}H-NMR spectrum of the reaction mixture after EChH of glutaraldehyde after baseline correction and peak deconvolution.

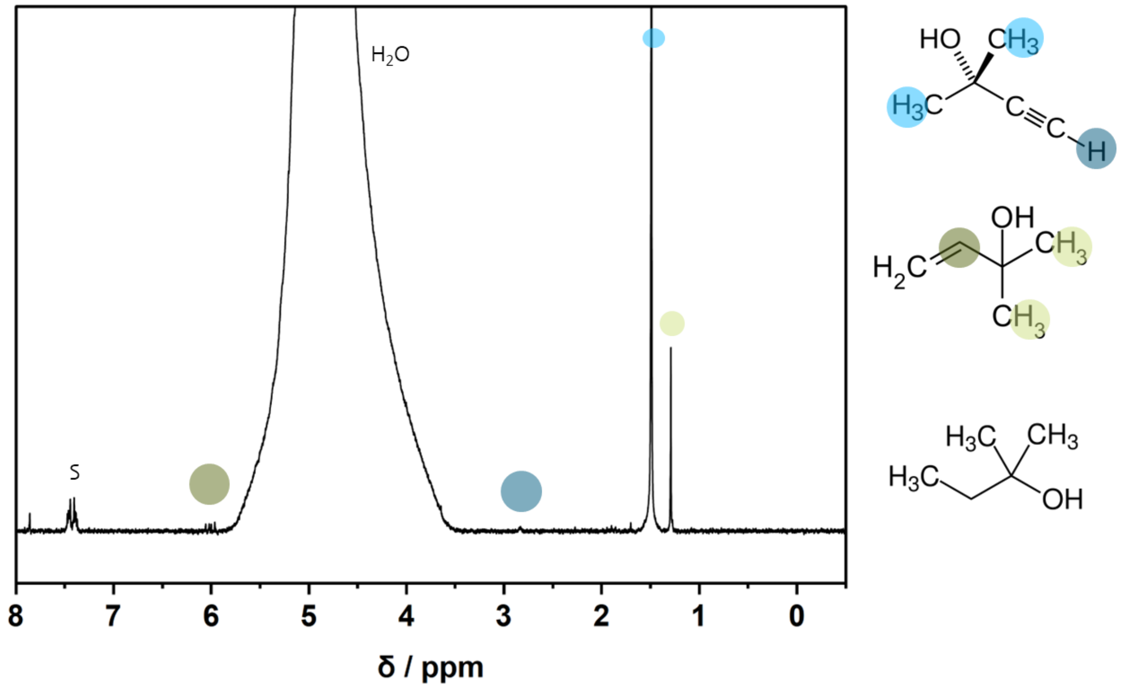

Figure S8: Representative ^{1}H-NMR spectrum of the anolyte solution after EChH of MBY after baseline correction and peak deconvolution.

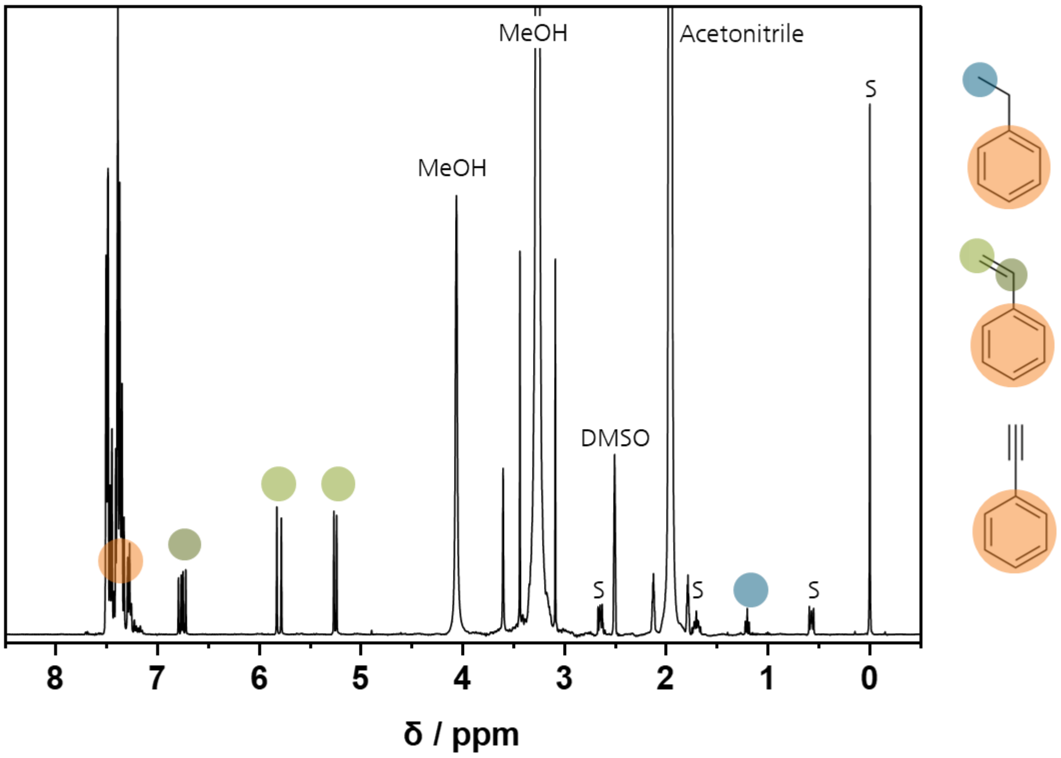

Figure S9.: Representative ^{1}H-NMR spectrum of the reaction mixture after EChH of phenylacetylene after baseline correction and peak deconvolution with d_3-acetonitrile.
